# Investigating psychometric properties and dimensional structure of an educational environment measure (DREEM) using Mokken scale analysis – a pragmatic approach

**DOI:** 10.1186/s12909-018-1334-8

**Published:** 2018-10-11

**Authors:** Per J Palmgren, Ulf Brodin, Gunnar H Nilsson, Roger Watson, Terese Stenfors

**Affiliations:** 1 Department of Learning, Informatics, Management and Ethics, Karolinska Institutet, SE-171 77 Stockholm, Sweden; 2 Department of Neurobiology, Care Sciences and Society, Karolinska Institutet, Stockholm, Sweden; 3 Faculty of Health & Sciences, University of Hull, Hull, England UK

**Keywords:** Dundee ready educational environment measure, Education, Educational measurement, Item response theory, Mokken scaling, Psychometrics, Undergraduate, Validity

## Abstract

**Background:**

Questionnaires and surveys are used throughout medical education. Nevertheless, measuring psychological attributes such as perceptions of a phenomenon among individuals may be difficult. The aim of this paper is to introduce the basic principles of Mokken scale analysis (MSA) as a method for the analysis of questionnaire data and to empirically apply MSA to a real-data example.

**Methods:**

MSA provides a set of statistical tools for exploring the relationship between items and latent traits. MSA is a scaling method of item selection algorithms used to partition an array of items into scales. It employs various methods to probe the assumptions of two nonparametric item response theory models: the monotone homogeneity model and the double monotonicity model. The background and theoretical framework underlying MSA are outlined in the paper. MSA for polytomous items was applied to a real-life data example of 222 undergraduate students who had completed a 50-item self-administered inventory measuring the educational environment, the Dundee Ready Educational Measure (DREEM).

**Results:**

A pragmatic and parsimonious approach to exploring questionnaires and surveys from an item response theory (IRT) perspective is outlined. The use of MSA to explore the psychometric properties of the Swedish version of the DREEM failed to yield strong support for the scalability and dimensional structure of the instrument.

**Conclusions:**

MSA, a class of simple nonparametric IRT models – for which estimates can be easily obtained and whose fit to data is relatively easily investigated – was introduced, presented, and tested. Our real-data example suggests that the psychometric properties of DREEM are not adequately supported. Thus, the empirical application depicted a potential and feasible approach whereby MSA could be used as a valuable method for exploring the behavior of scaled items in response to varying levels of a latent trait in medical education research.

## Background

Instruments such as questionnaires and surveys are used throughout medical education, including for the innumerable student evaluations of courses, programs, and clerkships as well as for student self-assessments and patient satisfaction. Moreover, survey-driven inquiries are extensively employed in medical education research [1]. A well-crafted questionnaire is a useful instrument for the measurement of underlying constructs or latent traits (variables).

Measurement has been defined as the process of applying numbers to objects in meaningful ways [2] and involves constructing a formal model of a dataset. Measurement and quantification is ubiquitous in many sciences. In social sciences, such as education and psychology, scholars are preoccupied with psychological measurements and concepts such as perceptions of and attitudes toward different phenomena. According to Bryman [3], there are three main reasons for the preoccupation with measurement in quantitative research: i) measurement allows researchers to delineate fine differences between people in terms of the phenomenon in question; ii) measurement provides a consistent device for making such distinctions; and iii) measurement provides for more precise estimates of the degree of relationships between phenomena. While systematic investigations of temperature and length with reliable measurements have been developed over centuries, systematic investigations into psychological measurements were undertaken only a century ago [4]. However, this is perhaps not merely a matter of a time lapse. Psychological measurement is inherently more difficult due to the properties being measured and does not lend itself equally straightforwardly to direct observation with a commonly accepted method. Measuring psychological attributes such as perceptions of a phenomenon among individuals can thus be difficult, albeit desirable.

### Survey scale design

Artino and colleagues [1] have highlighted some compulsory steps in the survey scale design process in medical education research: *conceptual elucidation of what is being explored, development and excerption of items, validation of content, substantiating item variance, reliability and convergent/discriminant validity with respect to other measures,* and *conclusive steps to scrutinize the construct validity of the survey/questionnaire.* This field of study which addresses many of these aspects is called psychometrics, and it is concerned with the theories, methods, and techniques of psychological measurement. In medical education research, there is a need for more stable and corroborated methods for interpreting and analyzing the results of questionnaires as well as possible new ways of drawing conclusions from such methods [5].

The methods employed to warrant the psychometric robustness of questionnaires can be placed on a continuum extending from common sense, item content, and choice of items to intricate mathematical and statistical models. However, it is a common misunderstanding that a questionnaire can be objectively validated, thus bearing good psychometric properties for diverse contexts [6]. Psychometric properties, such as validity, do not pertain to an instrument as such; rather, they are a feature of the construal of the results generated from a contextual study [7]. Therefore, when inventories are translated from a foreign language and/or applied to a different population, it becomes an empirical question, and findings need to be psychometrically scrutinized for the population in question. Otherwise, “It would be like visual observation using eyeglasses borrowed from someone else. It is bound to produce unclear or suboptimal results” [4].

It is common practice in medical education research (and other disciplines) to compose ordered items to sum scores in a questionnaire and to then use these sum scores for the corresponding statistics. Regardless of whether the intent is to create a sum score or any other aggregated measure for further use as a metric variable, we argue that this has to be explored. When generating an aggregated measure based on a set of items, such as a sum score, this entails establishing a model like any other statistical model, a model that has to be tested and its applicability examined. It is far from obvious that summarizing items in a questionnaire necessarily constitutes a valid continuous metric variable [5], and the problem with the assumption of item equivalence has been addressed in the literature [8]. Nevertheless, if a reasonable metric variable can be constructed, there are a variety of suitable psychometric methods available.

### Psychometric test theories

In many empirical studies in medical education research, the methods used to establish validity and reliability rely comprehensively on what is referred to as classical test theory (CTT), which includes methods such as principal component analysis and/or factor analysis for assessing the construct validity of scales, and/or internal consistency such as Cronbach’s alpha for the estimation of the reliability of test scores. These methods – which will not be elaborated on further in this article – deals with the estimation of measurement error and then forming, within the limits of the methods available, an estimate of the true score. CTT relies mostly on the assumption of continuous data and commonly a normal distribution of data and mainly investigates the relationship between items and total scale scores. Further, in CTT, the scale score is not very informative about the item response pattern, and any combination of scores on any set of items can give the same score on the latent trait. As such, van Schuur [9] has suggested that CTT may have limited insight as to whether sets of items measure the same concept.

An alternative method to CTT is item response theory (IRT), which pursues much of the same problems as CTT and was developed particularly for nominal and ordinal questionnaire data. Further, IRT can often be a supplement to CTT in terms of detecting sets of items that measure the same concept [9]. IRT also augments interpretive power by establishing measurement precision that is distinct with a person’s ability level [10]. Thus, this data (e.g., an error that fluctuates based on person performance) can be utilized to distinguish weak and critical parts of a questionnaire under scrutiny [11]. There are a multitude of IRT methods and techniques, however, it is beyond the scope of this paper to address them. One branch of the IRT method is Mokken scale analysis (MSA or Mokken scaling), which is based on the principles of IRT and a scaling method proven to be valuable for assessing the psychometric properties of questionnaire data [4, 12–14].

Taking the above factors into account, the aim of this paper is: 1) to introduce the basic principles of MSA as a method for the analysis of questionnaire data; 2) to provide a pragmatic and parsimonious approach to explore questionnaires from an IRT perspective; and 3) to empirically apply MSA to a real-data example.

## Methods

### Basic principles of MSA

**Fig. 1:**
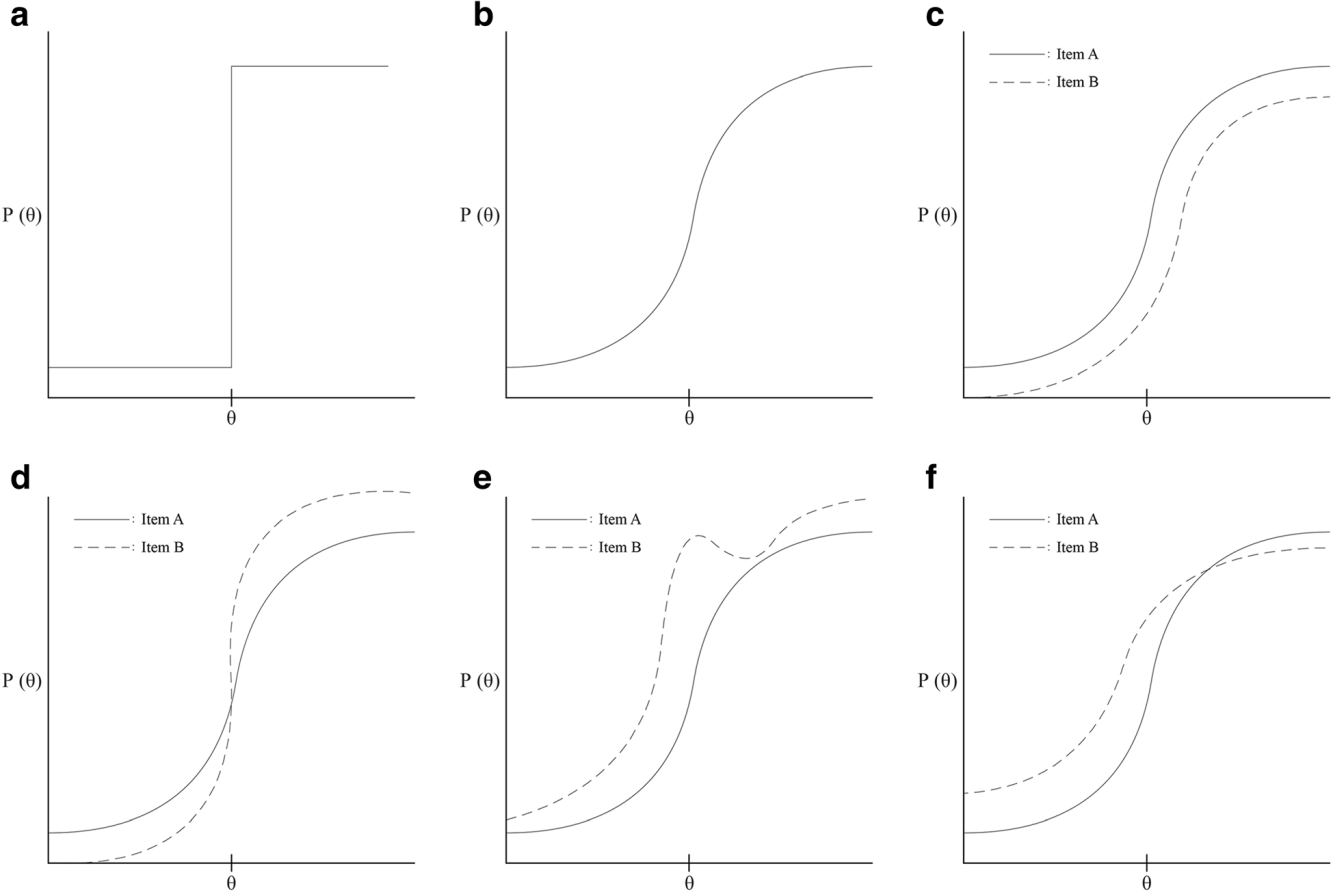
Various Item response functions (IRF). (**a**) Depicting an item performing according to the deterministic Guttman model. (**b**) An example of an item performing in a stochastic manner. (**c**) Two items displaying distinct levels of difficulty (item B more difficult than item A). (**d**) Two items depicting distinct levels of discrimination (item B more discriminating than item A). (**e**) Two items displaying MHM and where Item B is monotonically decreasing, thus violating MHM. (**f**) Two items exhibiting DMM and where Item B intersects Item A and confirms invariant item ordering, thus violating DMM

MSA is an analytical method that provides a set of statistical tools for exploring the reciprocity and relation between items and latent traits. It evolved from the Guttman scaling model, which is based on the assumption that the items in a scale are hierarchically ordered: this means that they are ordered by their degree of “difficulty,” difficulty referring to the ease and extent with which an item is endorsed by respondents (See Watson et al. [15] for a more comprehensive discussion). Thus, Guttman scaling model is deterministic as it does not allow for the possibility of any stochastic elements. It does not regard the relation between an item and the latent trait in terms of probability. Rather, it is discriminatory of the latent trait on the basis of the endorsement, or lack thereof, of an item. Figure 1a displays an example of an item behaving in consonance with the deterministic Guttman scaling model along a latent trait on the X-axis with the probability of an affirmative response to the item on the Y-axis.

#### Item response function

Central to the models derived from IRT (such as MSA) is how discrete items in a scale perform in relation to the latent trait, which can be described by an item response function (IRF) or an item characteristic curve [16]. IRFs can be regarded as the fundamental unit of analysis in IRT methods. They describe the relationship between the latent trait and the probability of responding positively to that item, whereby “positively” means endorsing the item in attitudinal scales (or answering correctly in ability scales). The trait level is signified by theta (denoted as θ), and the IRF for a specific item represents the probability – Р (θ) – of an individual’s item score being acquired in the presence of a specific level of the latent trait. In general, the IRF echoes the notion that the higher the latent trait value of θ, the higher the probability of a score on the item that measures θ, consequently increasing non-linearly. Figure 1b shows an item responding stochastically in the presence of θ on X-axis and Р (θ) on the Y-axis as IRT models attempt to fit the data to sigmoid-shaped curves IRFs can differ according to item difficulty. Figure 1c depicts the IRFs for two items, where the probability of endorsing one item (Item A) is more noteworthy than the probability of endorsing the other (Item B), consequently the item (B) being more difficult. IRFs can also display item discrimination, a measure of the differential capability of an item. A high discrimination suggests that an item has a high ability to differentiate subjects. As shown in Fig. 1d, the pitch of the IRFs can be assessed, as items with a greater slope (Item B) can be regarded as more discriminatory than those whose slope is shallower (Item A).

### A non-parametric item response theory model

MSA is a data reduction method aimed at assessing unidimensional scales of dichotomous (binary) or polytomous (ordinal) items and belongs to the class of non-parametric item response theory (NIRT). MSA can be applied when designing or constructing multi-item questionnaires; as a secondary analysis to more well-established CTT or parametric IRT methods (PIRT); or to investigate the conformity and consonance of new data in which well-known items are applied to new group of respondents [12]. MSA also has some advantages over PIRT models, such as the Rasch model. First, MSA depends on less restrictive assumptions and is less demanding on the data, while maintaining important measurement properties, which prevents researchers from unnecessarily removing items from a scale. Second, MSA provides valuable tools for exploratory dimensionality analyses that are not readily available for PIRT models [17, 18]. Further, it has been postulated that “before assessing the possibility of using a sum score as a sufficient statistics to establish a reasonable ‘person measure’ on an interval scale, an initial step would be to gauge data by means of a non-parametric approach” [6].

#### Assumptions underlying NIRT models

At the beginning of the 1970s, Robert Mokken proposed his thesis of two NIRT models for dichotomous items [14]: the monotone homogeneity model (MHM) and the double monotonicity model (DMM) to underpin MSA, a scaling technique for ordinal data. Nearly ten years later, Molenaar [19] developed these models to cater for polytomous items (more comprehensive discussions of these models can be found in [4, 20, 21].

MSA can be applied in a confirmatory manner, for a set of items that are assumed to form a scale, or in an exploratory manner when a set of items is analyzed to ascertain whether it constitutes one or more scales. Both confirmatory and exploratory approaches employ the same criteria, the only differences being what is entered into the analysis and the assessment of whether the clusters of items (dimensions), which are found or tested, adhere to one or two NIRT models. These models are grounded in four assumptions that must be met in order to endorse and stipulate Mokken modeling. These assumptions are: *unidimensionality, monotonicity, local independence,* and *invariant item ordering (IIO)* [13, 22].

The assumption of unidimensionality means that for those items forming a scale, there is a prevailing single latent trait (θ) that governs the answers to the items [23]. Unidimensionality is commonly considered a desirable measurement property because it simplifies the interpretation of answers to the items and averts the total score of the items from expressing a potpourri of different traits. However, unidimensionality does not mean that it is impossible for more than one dimension to exist in a large set of items; rather, that clusters of items fitting an NIRT model are unidimensional.

The second assumption, monotonicity, alludes to the increasing probability of the score on an item increasing as the level of the latent trait increases; thus, the endorsed response P (θ) is a monotonically non-decreasing function of the latent trait θ. Figure 1e exhibits one item (A) increasing monotonically and one item (B) which is not, thus indicated by a slight dip in the IRF. Aberrations from this premise indicate violations of monotonicity and conceivable distortions from and misuse of ordinal scale for measuring persons.

The assumption of local independence stipulates that a person’s responses to items on a scale are reliant on his or her level on the latent trait being measured; the response to one item is not influenced and affected by the score on any other [24]. It should be emphasized that this is largely a conjecture, as utter local stochastic independence is virtually undetectable and practically unachievable [12, 15].

The three aforementioned assumptions are adequate for numerous NIRT procedures and encompass the assumptions of the MHM. The more limiting DMM necessitates the additional assumption of non-intersecting of IRFs traversing θ. Thus, non-intersecting IRFs is confirmed by invariant item ordering and refers to items on a scale with the same level of “difficulty” in terms of ordering across all respondents at all levels of the latent trait. This is shown in Fig. 1f where Item B intersects with Item A, thus Item B violates the DMM. The IIO property is decisive in establishing hierarchical scales. If these four assumptions are not excessively violated, higher sum scores are seen as corresponding to higher values on the latent trait, suggesting that respondents can be reliably ordered on the latent trait by their sum scores. By retaining a “bottom up” clustering technique by means of preselected cut-off values for item scalability, MSA permits analyses of the dimensional structure of a scale or scales on different hierarchical levels [15, 25].

### MSA as a pragmatic and parsimonious approach

**Fig. 2:**
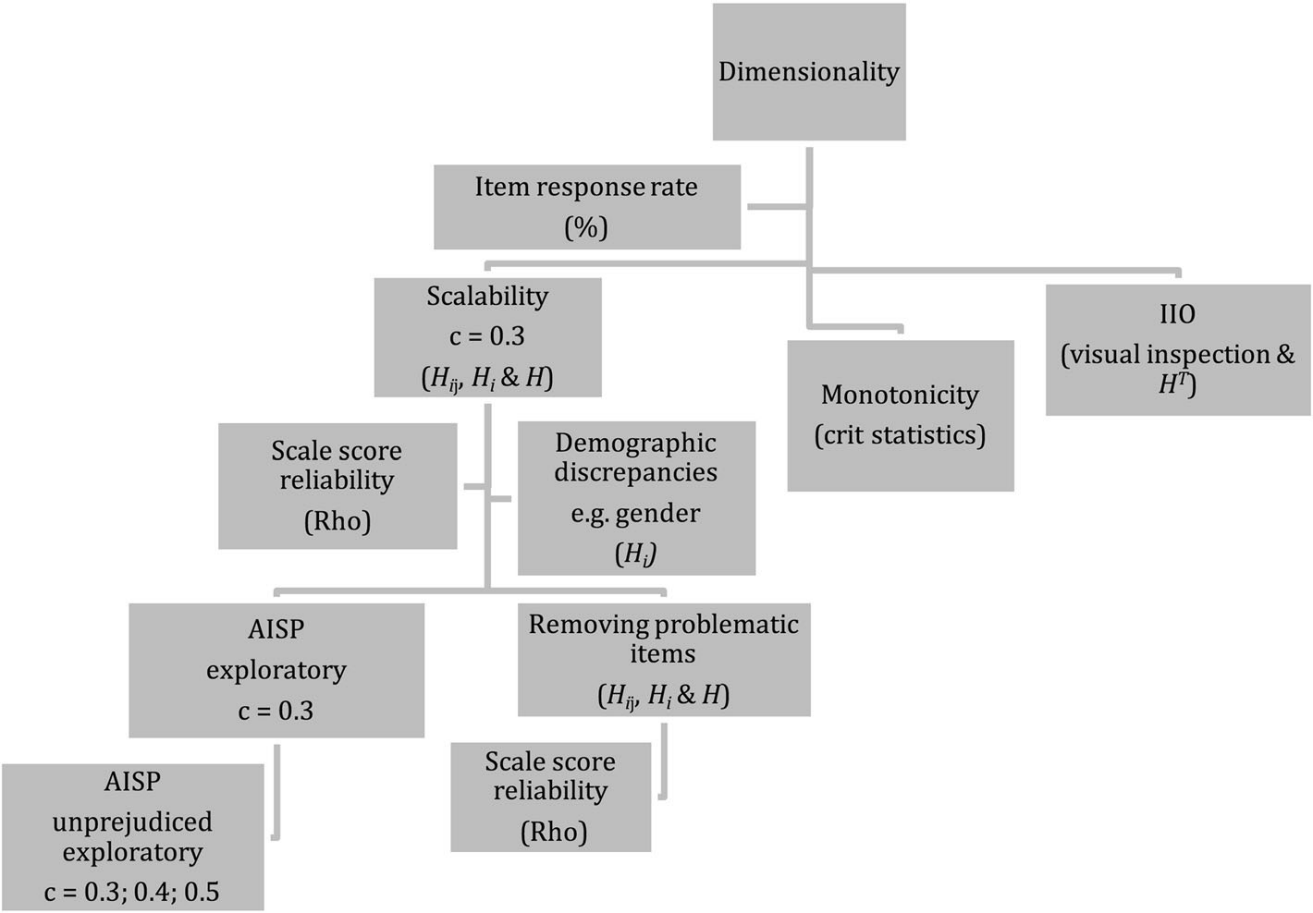
Investigation approach and analytic procedure. The figure exhibits a pragmatic and parsimonious approach to MSA incorporating a number of sequential steps

As shown in Fig. 2 we propose a pragmatic and parsimonious approach to MSA, which incorporates several sequential steps. Despite the fact that the ensuing steps depicted in the figure might seem consecutively ordered, the pragmatic analytical approach, is not linear, but iterative and recursive. A well-known dilemma in data analysis during questionnaire testing is that some respondents do not provide answers to some of the items in a scale, resulting in an incomplete data matrix [26]. Consequently, a few non-systematic missing values might be imputed, e.g., using a two-way imputation or a hot deck imputation, thus replicating values from other respondents with analogous but comprehensive response patterns in order to make full use of the sample. However, we argue and concur with Brodin [6] that missing values in questionnaires should in general not be subject to imputation as the respondent has chosen not to answer. Subsequently, data are not missing as empty cells signify “no response” rather than “missing data”. Further, there is drawback applying imputations in Mokken scaling as discussed by van der Ark and Sijtsma [27] who show, using simulation methods that, while there is little to choose between methods of imputation, all lead to clusters of items that deviate from the original solutions without missing data. Thus, we recommend that inventories containing “no response” to any item should be discarded from the analysis as MSA focuses on the multiple and partial relationship (scalability) between items, and that no collapsing of categories is performed.

In exploring data in relation to the item response rate (IRR) the floor and ceiling effects can be examined. IRR can be explored as the proportion of respondents completing all inventorial items, with a level of ≥90% considered an acceptable threshold [28]. In order to investigate floor or ceiling effects – people obtaining minimum and maximum scores, respectively – descriptive statistics can be used, assessing proportions and arbitrarily considering a poor result to be more than 20% of respondents recording the minimum or maximum score [8].

MSA works by pursuing unidimensional sets of items based on Loevinger’s definition of homogeneity and the coefficient *H* [29] and the extent to which pairs of items, as scored by respondents, conform. Homogeneity is sometimes confused with internal consistency, the former denoting the unidimensionality of a measure, while the latter refers to the degree of interrelatedness among items in a measure [30]. Mokken scales are demarcated by means of scalability coefficients [14], and the first part of MSA involves the testing of hypotheses about these scalability coefficients, three of which are indispensable: 1) For each pair of items, *i* and *j*, there is an *item pair-scalability coefficient*, denoted as *H*_*ij*_, which can be attributed, in very simple terms, as the covariation between two ordered variables. 2) Analogous to the pairwise coefficient, there is also an *item scalability coefficient*, designated as *H*_*i*_, articulating how much an item is correlated to the sum score based on the remaining set of variables. 3) For the complete set of items, there is a *test scalability coefficient*, denoted as *H*, conveying the degree to which the total scores accurately rank persons on the latent trait. The common practice for interpreting dimensionality by means of coefficient *H* is: a scale is considered strong when estimate *H ≥* 0.5, moderate 0.4 *≤ H <* 0.5, and weak 0.3 *≤ H <* 0.4. If the scalability is found to be insufficient, 0 < *H* < 0.3, it is considered very weak, and the sum score approach might be discarded as unsuitable, suggesting that the instrument produced scarce or negligible information [4]. A scale of *H* < 0 is considered counterproductive and warrants deletion of an item/items. A scalability analysis of data can also be employed to scrutinize whether the items and scale structure differs regarding demographic or other variables such as gender, age, year of class. These demographic variations can be introspected visually by plotting *H*_*i*_ coefficients for the intended variables.

Monotonicity and IIO are subsequently investigated. Monotonicity is assessed through the number of violations of the assumption, and the seriousness of these violations was evaluated by means of *crit* statistics. *Crit* statistis [31] are a weighted sum of other components (i.e., *H*_*i*_, the number of possible and actual violations in which the item can be involved) and may be used for evaluating monotonicity [13]. According to Stochl et al. [12], items for which the *crit* statistic is < 40 do not seriously violate the criteria for monotonicity and may therefore harmlessly be included in the Mokken scale. IIO can be visually assessed by plotting item pairs and scrutinizing for the non-intersection of item characteristic curves. A comparable coefficient to Loevinger’s *H* coefficient called *Htrans* (*H*^*T*^) can also be employed; the range of values of *H*^*T*^ can be interpreted as follows: 0.3 ≤ *H*^*T*^ < 0.4 designating weak IIO; 0.4 ≤ *H*^*T*^ < 0.5 and *H*^*T*^ ≥ 0.5 indicating moderate and strong IIO, respectively [32, 33].

Once a scale has been finalized *Mokken’s Rho* is used to estimate the score reliability, the values of which should exceed 0.70 [4, 34]. This method was developed specifically in the context of MSA—described as an unbiased estimator of reliability—which is considered to be an improvement in relation to Cronbach’s alpha (see Molenaar and Sijtsma [34] for a more elaborated explanation of the mathematical equation related to *Mokken’s Rho* also known as the Molenaar–Sijtsma method).

In the subsequent step, an exploratory analysis can be employed using an MSA feature called the automated item selection procedure (AISP) to find Mokken scale solutions from a set of items such as a subscale. First, a set of items forms a so-called Mokken scale if two conditions are met: (1) for all item pairs, scalability coefficient *H*_*ij*_ is > 0, and (2) scalability coefficient *H*_*i*_ is greater than some a priori chosen lower-bound discrimination threshold. This minimum scalability threshold coefficient is also known as the user-specified constant, c. A lower-bound value c is optional, although its default value equals 0.3. Following this, additional items are selected in sequential order on the basis of the following criteria: (1) the item correlates positively with the formerly selected items; (2) its scalability coefficient with respect to the designated items is > 0 and surpasses the user-specified constant; and (3) the accumulation of the item produces the largest scalability coefficient of all the items that could have been selected. When there are no surplus items that meet these criteria, a new iteration begins using the remaining unselected items. The AISP ends when all items in the pool have been partitioned into a Mokken scale or when none of the remaining items meet the discrimination criteria. This procedure can be concluded with an unprejudiced exploratory analysis, leaving the AISP completely free to establish Mokken scale solutions from the entire item inventory pool.

#### Software for NIRT analysis

Several software programs are currently available for data analysis using non-parametric IRT. The Mokken scaling procedure (MSP) is commercially accessible for Windows [31], and there is also a module in the statistical software Stata [35]. Test Graf is a public domain software for investigating item properties [36], and the free right to property software R also contains Mokken scaling analysis [13, 37].

### Empirical application of MSA

One of the benchmarks for measuring the undergraduate educational environment is the Dundee Ready Educational Environment Measure (DREEM), with items allocated around an a priori five-factor model [38]. Although the DREEM instrument was initially reported to have good construct validity in its original contexts [38, 39], more recently, investigators have impugned the psychometric properties—internal consistency and construct validity—of the measure, asserting that the model itself may be in need of revision [28, 40–43]. Reproductions of the original scale structure have only been moderately successful, perhaps indicating weaknesses in the instrument, and some contradictory evidence exists in the scholarly literature [28, 42, 44]. Researchers have also advocated caution when calculating the overall sum score as the instrument has been unable to gauge a single underlying construct [43].

#### Setting

The study was conducted at the Karolinska Institutet, a medical university in Stockholm, Sweden.

#### Participants

A convenience sample consisting of undergraduate physiotherapy students from five terms (T1–T5) attending a traditional curriculum was employed. The DREEM inventory was administered during classes to ensure a high response rate. However, an electronic version of the inventory was subsequently disseminated to improve the response rate. Completion of the DREEM inventory was undertaken on a voluntary basis, and no identifiable information was collected, thus maintaining data anonymity.

#### Measure

DREEM is a self-administered, closed-ended inventory relating to a variety of topics of direct relevance to educational environments. It has been translated for use in Sweden [28]. The DREEM inventory comprises 50 statements, which are gradually scored from 0 to 4. The response alternatives are: 0 = strongly disagree, 1 = disagree, 2 = unsure, 3 = agree, and 4 = strongly agree, thus constituting an ordinal scale. This is often referred to, incorrectly or otherwise [45, 46], as a Likert scale. The items are congregated into five subscales: students’ perceptions of learning (SPL-12 items/maximum score 48), students’ perceptions of teaching (SPT-11 items/maximum score 44), students’ academic self-perceptions (SASP-8 items/maximum score 32), students’ perceptions of atmosphere (SPA-12 items/maximum score 48), and students’ social self-perceptions (SSSP-7 items/maximum score 28). The instrument has an overall score of 200. Nine items are negative statements and are therefore scored in reverse.

#### Statistical procedure

In our dataset, inventories containing “no response” to any item were discarded from the analyses, no imputations were applied, and no collapsing of the categories was performed. DREEM items that are negatively stated were recoded so that for all items, higher scores would mean a higher position on the attribute scale. Data were entered into the Statistical Package for the Social Sciences (SPSS) version 22.0 database and converted into a format suitable for MSA in Mokken package R 3.0.3 (R Development Core Team 2011).

## Results

### Inventory response rate

Of a total population of 278 students from five terms, 222 students completed the inventory, thereby yielding an overall response rate of 80%. The respondents included 169 female (76%) and 53 male (24%) students. The mean age was 24.7 (median 23; interquartile range (IQR) 21–26; range 19 and 52) years.

### Item response rate

Thirty-nine participants (18%) did not complete all fifty items, and the number of non-responses for each item ranged between 1 (0.5%) and 14 (6.3%). Items 6 (*n* = 13, 5.9%) and 18 (*n* = 14, 6.3%) displayed the highest proportion of internal non-responses, and the analysis revealed that these omitted responses were mainly from students in terms 1 and 2. By discarding these two items, the non-responses ranged between 1 (0.3%) and 6 (1.5%). The frequency of non-responses in the subscales (all items incorporated) included SPL: 0.9%; SPT: 14.9%; SASP: 5.9%; SPA: 5.4%; and SSSP: 0.5%. No floor effects were observed in the data, and only minor ceiling effects were identified for SPL, SPT, SASP, and SPA, ranging between 0.5 and 1.4%. SSSP displayed the largest ceiling effect, with 10 respondents (4.5%) scoring the maximum.

### Scalability assessment

**Fig. 3:**
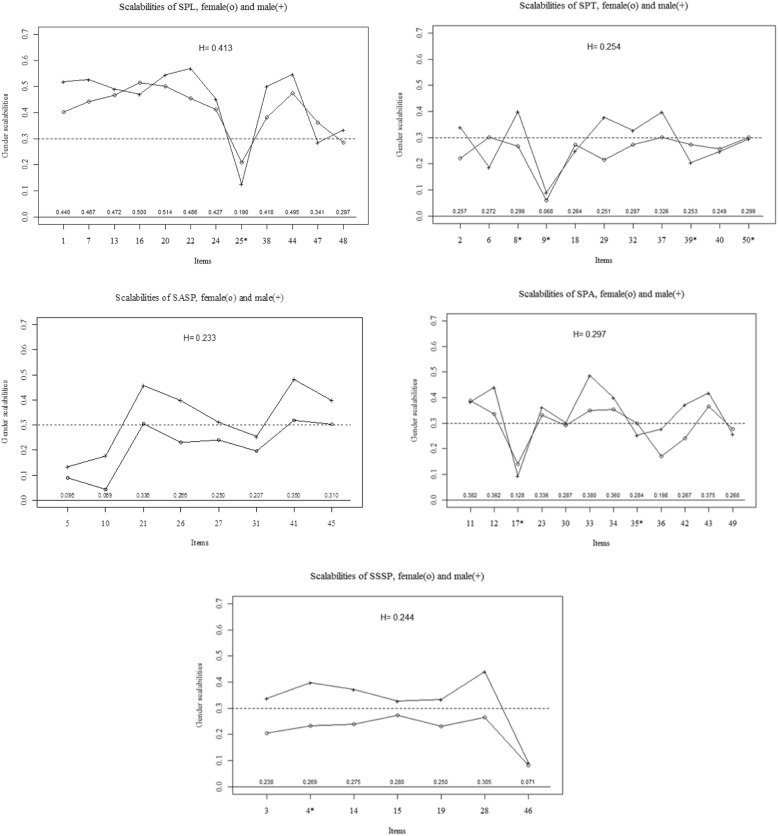
Gender variations. The graph illustrates gender variations in item scalabilities for subscales: students’ perceptions of learning (SPL); students’ perceptions of teaching (SPT); students’ academic self-perceptions (SASP) students’ perceptions of the atmosphere (SPA); and students’ social self-perceptions (SSSP)

The item pair scalability (*H*_*ij*_) for SPL ranged from 0.003 to 0.384. Item 25 had a low scalability with many of the other items. The scale showed moderate scalability (*H* = 0.413), as most of the items contributed to the intended dimension. As indicated in Table 1, two reversed items (25 and 48) were weak but related to each other (*H*_*ij*_ = 0.384). No major gender variations were observed, as visually displayed in Fig. 3.

**Table 1:** Discrete item scalabilities in relation to the subscales

SPL^1^	Item	1	7	13	16	20	22	24	25^a^	38	44	47	48^a^
	*H*_*i*_	.440	.467	.472	.500	.514	.486	.427	.190	.418	.495	.341	.297
SPT^2^	Item	2	6	8^a^	9^a^	18	29	32	37	39^a^	40	50^a^	
	*H*_*i*_	.257	.272	.299	.068	.264	.251	.287	.326	.253	.249	.299	
SASP^3^	Item	5	10	21	26	27	31	41	45				
	*H*_*i*_	.096	.069	.336	.266	.250	.207	.350	.310				
SPA^4^	Item	11	12	17^a^	23	30	33	34	35^a^	36	42	43	49
	*H*_*i*_	.382	.362	.128	.336	.287	.380	.360	.284	.198	.267	.375	.268
SSSP^5^	Item	3	4^a^	14	15	19	28	46					
	*H*_*i*_	.238	.269	.275	.280	.250	.305	.071					

Abbreviations: *SPL* students’ perceptions of learning, *SPT* students’ perceptions of teaching, *SASP* students’ academic self-perceptions, *SPA* students’ perceptions of the atmosphere, and *SSSP* students’ social self-perceptions. A superscript letter indicates *H* coefficient for the subscale: ^1^0.413, ^2^0.254, ^3^0.233, ^4^0.297 and ^5^0.244

^a^Indicates negatively stated items

In the SPT scale, *H*_*ij*_ ranged from − 0.018 to 0.583. Reversed item 9 revealed negative scalability with items 6 and 37. The *H* value of 0.254 indicated a very weak scalability with item 37 (*H*_*i*_ = 0.325), only surpassing the threshold of c > 0.3 (Table 1). The reversed item 9 had a negligible relationship with other reversed items (8, 39, and 50). As shown in Fig. 3 male students recorded marginally higher *H*_*i*_ values and item 29 portrayed some indicative variation.

The SASP scale contained no reversed items, but several items displayed negative relationships, especially items 5 and 10. The analysis yielded insufficient scalability (*H* = 0.233), and items 5 (*H*_*i*_ = 0.096) and 10 (*H*_*i*_ = 0.069) showed no relationship with the remainder of the items or the SASP dimension (Table 1). As illustrated in Fig. 3 male and female students showed similar response structures, but with the latter displaying consistently higher *H*_*i*_ coefficients.

For the SPA subscale, the item pair scalabilities ranged from − 0.060 to 0.675. Reversed item 17 displayed virtually no scalability with items 36 and 42 (both *H*_*i*_ = − 0.060). As indicated in Table 1, the SPA showed weak scalability (*H* = 0.297). In particular, reversed item 17 displayed weakness (*H*_*i*_ = 0.128). As exhibited in Fig. 3 no obvious gender discrepancies were observed.

Regarding the dimension of SSSP, *H*_*ij*_ ranged from − 0.003 to 0.385, with item 46 indicating no scalability with items 14 (*H*_*ij*_ = − 0.003) and 28 (*H*_*ij*_ = − 0.014). As illustrated in Table 1, the *H* value of 0.244 indicated insufficient scalability, and the value of item 46 was particularly low (*H*_*i*_ = 0.071). As displayed in Fig. 3, while male and female respondents reported similar response tendencies, the male respondents reported mostly above the threshold.

### Monotonicity and IIO

In the SPL subscale, item 25 showed some (*crit* = 55), though not significant, violations against monotonicity. For the SPT, items 9 and 40 displayed very high *crit* values: *crit* = 126, and *crit* = 73, respectively. Item 10 in the SASP subscale exhibited a *crit* value of 88, thus indicating a violation of monotonicity. For the SPA subscale, item 30 exhibited a *crit* value of 40, however, this was not significant, and a violation of monotonicity could not be statistically demonstrated. In the SSSP dimension, item 46 exhibited a tendency of high *crit* values but did not surpass the threshold of > 40.

Item pair plots showed that while there were no intersecting items (which would indicate violation of IIO) and that most of the item characteristic curves for the individual scales clustered together, a few item characteristic curves lay at some distance from the rest, for example, items 9 and 36. In the assessment of IIO by means of *H*^*T*^, the SPL *(H*^*T*^ = 0.41) and SPT (*H*^*T*^ = 0.46) subscales demonstrated moderate IIO. The IIOs for the SASP and SPA dimensions, *H*^*T*^ = 0.20 and *H*^*T*^ = 0.16, respectively, indicated that the order of the items was not invariant over the two latent scales, thus violating the assumption of IIO. The IIO for the SSSP subscale (*H*^*T*^ = 0.33) was considered weak.

### Scale score reliability

As portrayed in Table 2, the *rho* estimates for the SPL and SPA subscales were good. The score reliability estimates for the SPT dimension were fair but surpassed the recommended value of 0.70 (Table 2). However, the score reliabilities for the SASP and SSSP subscales were low.

**Table 2:** *Rho* coefficients (Molenaar–Sijtsma method) as estimates of the score reliability for the subscales

Subscales	SPL	SPT	SASP	SPA	SSSP
Original	.866	.763	.675	.812	.659
(No. of items)	(12)	(11)	(8)	(12)	(7)
Problematic items deleted	.883	.770	.744	.816	.667
(No. of items)	(10)	(7)	(6)	(10)	(5)

Abbreviations: *SPL* students’ perceptions of learning, *SPT* students’ perceptions of teaching, *SASP* students’ academic self-perceptions, *SPA* students’ perceptions of the atmosphere, and *SSSP* students’ social self-perceptions

### Exploratory AISP

As presented in Table 3, an exploratory AISP on the items of the SPL dimension generated two scales (*H* = 0.513 and *H* = 0.384), with the second comprising two reversed items: 25 and 48. The exploratory evaluation of the SPL scale generated three subscales. Three of the four reversed items (8, 39, and 50) produced one common scale (*H* = 0.535), while the remaining item (9) was not scalable (Table 3). The AISP partition of the items belonging to SASP generated one scale (*H* = 0.412), however, items 5, 10, and 31 were distinguished as unscalable (Table 3). The items belonging to the SPA dimension were divided into two subscales, one containing nine items (*H* = 0.379) and the other comprising only two items (*H* = 0.336). Reversed item 17 was designated as unscalable (Table 3). The SSSP items were partitioned into two subscales (*H* = 0.417 and *H* = 0.336), with an inability to assign item 46 to any scale.

**Table 3:** Scalabilities after exploratory dimensionality analysis by automated item selection procedure

	SPL			SPT			SASP			SPA			SSSP	
	Item		*H*_*i*_	Item		*H*_*i*_	Item		*H*_*i*_	Item		*H*_*i*_	Item	*H*_*i*_
*Scale 1* *H = .513*	1	.515	*Scale 1* *H = .400*	6	.377	*Scale 1* *H = .412*	21	.486	*Scale 1* *H = .379*	11	.446	*Scale 1* *H = .417*	15	.432
	7	.528		18	.396		26	.342		12	.399		19	.375
	13	.508		29	.426		27	.324		23	.408		28	.437
	16	.571		32	.408		41	.468		30	.322			
	20	.580		37	.388		45	.450		33	.427	*Scale 2* *H = .336*	3	.319
	22	.541								34	.435		4^a^	.314
	24	.516	*Scale 2* *H = .535*	8^a^	.544					35^*^	.308		14	.375
	38	.470		39^a^	.546					43	.383			
	44	.566		50^a^	.513					49	.322			
	47	.370												
			*Scale 3* *H = .373*	2	.373				*Scale 2* *H = .366*	36	.366			
*Scale 2* *H = .384*	25^a^	.384		40	.373					42	.366			
	48^a^	.384												
			Item 9^a^ non-scalable			Items 5,10 & 31 non-scalable			Item 17^a^ non-scalable			Item 46 non-scalable		

Abbreviations: *SPL* students’ perceptions of learning, *SPT* students’ perceptions of teaching, *SASP*, students’ academic self-perceptions; SPA, students’ perceptions of the atmosphere; and SSSP, students’ social self-perceptions

^a^Indicates negatively stated items

#### Unprejudiced exploratory AISP

The entire DREEM inventory was exposed to an unprejudiced exploratory Mokken analysis to investigate whether the five predefined subscales could be retrieved and to determine how close they were to each other. This unprejudiced AISP could be regarded as very tolerant exploratory analysis, thus leaving the AISP inhibited to aggregate scale solutions from the item inventory pool. The analysis generated *H* = 0.264 for all fifty items, indicating multidimensionality. As presented in Table 4, using the AISP at a lower bound of c = 0.3 revealed clustering of items around six dimensions. The majority of items were clustered to the first dimension and seven (14%) items were not scalable (Table 4). Table 5 is an extension of Table 4 and displays in greater detail the assigned items from the first dimension using the threshold’s lower bound. This tolerant exploratory analysis was also performed when increasing the lower bound, c = 0.4, which yielded six dimensions, with 32% of the items unallocated. Increasing the lower bound to c = 0.5 generated five dimensions, with 44% of the items being non-scalable. A similar unprejudiced exploratory analysis was performed with 38 items, resulted from the removal of twelve problematic items. No negative relationships were detected, and the 38-item scale generated an *H* value of 0.354. Four dimensions were generated using the AISP, with a lower bound of 0.3 and two items as non-scalable (results not reported).

**Table 4:** Dimensionality of items from unprejudiced exploratory automated Item selection procedure

Item number and phraseology		Dimensions from AISP c = 0.3						
	*H*_*i*_	1	2	3	4	5	6	Not scalable
1. I am encouraged to participate in class	.352	SPL1						
2. The teachers are knowledgeable	.266	SPT2						
3. There is a good support system for students who get stressed	.291	SSSP3						
4. I am too tired to enjoy this course^a^	.278	SSSP4						
5. Learning strategies which worked for me before continue to work for me now	.089							SASP5
6. The teachers are patient with the patients	.251	SPT6						
7. The teaching is often stimulating	.373	SPL7						
8. The teachers ridicule the students^a^	.195		SPT8					
9. The teachers are authoritarian^a^	.001							SPT9
10. I am confident about my passing this year	.060							SASP10
11. The atmosphere is relaxed during the clinical teaching	.302	SPA11						
12. This school is well timetabled	.306	SPA12						
13. The teaching is student centered	.360	SPL13						
14. I am rarely bored in this course	.346	SSSP14						
15. I have good friends in this school	.201				SSSP15			
16. The teaching helps to develop my competence	.384	SPL16						
17. Cheating is a problem in this school^a^	.076							SPA17
18. The teachers have good communication skills with patients	.291	SPT18						
19. My social life is good	.223				SSSP19			
20. The teaching is well focused	.394	SPL20						
21. I feel I am being well prepared for my profession	.350	SASP21						
22. The teaching helps to develop my confidence	.389	SPL22						
23. The atmosphere is relaxed during lectures	.268	SPA23						
24. The teaching time is put to good use	.336	SPL24						
25. The teaching overemphasizes factual learning^a^	.175					SPL25		
26. Last year’s work has been a good preparation for this year’s work	.203							SASP26
27. I am able to memorize all I need	.237						SASP27	
28. I seldom feel lonely	.296	SSSP28						
29. The teachers are good at providing feedback to students	.247	SPT29						
30. There are opportunities for me to develop interpersonal skills	.296	SPA30						
31. I have learned a lot about empathy in my profession	.207							SASP31
32. The teachers provide constructive criticism here	.293	SPT32						
33.I feel comfortable in class socially	.294	SPA33						
34. The atmosphere is relaxed during seminars/tutorials	.237			SPA34				
35. I find the experience disappointing^a^	.318	SPA35						
36. I am able to concentrate well	.196						SPA36	
37. The teachers give clear examples	.326	SPT37						
38. I am clear about the learning objectives of the course	.325	SPL38						
39. The teachers get angry in class^a^	.163		SPT39					
40. The teachers are well prepared for their classes	.260	SPT40						
41. My problem-solving skills are being well developed here	.375	SASP41						
42. The enjoyment outweighs the stress of studying physiotherapy	.335	SPA42						
43. The atmosphere motivates me as a learner	.409	SPA43						
44. The teaching encourages me to be an active learner	.413	SPL44						
45. Much of what I have to learn seems relevant to a career in physiotherapy	.365	SASP45						
46. My accommodation is pleasant	.069							SSSP46
47. Long-term learning is emphasized over short-term learning	.239	SPL47						
48. The teaching is too teacher centered^a^	.238					SPL48		
49. I feel able to ask the questions I want	.241			SPA49				
50. The students irritate the teachers^a^	.190		SPT50					
	.264^b^	.406^b^	.554^b^	.402^b^	.398^b^	.370^b^	.360^b^	.048^b^

Abbreviations: *AISP* automated item selection procedure, *SPL* students’ perceptions of learning, *SPT* students’ perceptions of teaching, *SASP* students’ academic self-perceptions, *SPA* students’ perceptions of the atmosphere, and *SSSP* students’ social self-perceptions

^a^Indicates negatively stated items

^b^Designates test scalability coefficient (*H*)

**Table 5:** Suggested allocations from the first dimension based on unprejudiced exploratory automated item selection procedure

Item *H*_*i*_ (founded on 50 items)	First dimension from AISP		
	c = 0.3		
.350	SASP21		
.375	SASP41		
.365	SASP45		
.302		SPA11	
.306		SPA12	
.268		SPA23	
.296		SPA30	
.294		SPA33	
.318		SPA35^a^	
.335		SPA42	
.409		SPA43	
.352			SPL1
.360			SPL13
.384			SPL16
.394			SPL20
.389			SPL22
.336			SPL24
.325			SPL38
.413			SPL44
.239			SPL47
.373			SPL7
.291	SPT18		
.266	SPT2		
.247	SPT29		
.293	SPT32		
.326	SPT37		
.260	SPT40		
.251	SPT6		
.346		SSSP14	
.296		SSSP28	
.291		SSSP3	
.278		SSSP4^a^	

Abbreviations: *AISP* automated item selection procedure, *SPL* students’ perceptions of learning, *SPT* students’ perceptions of teaching, *SASP* students’ academic self-perceptions, *SPA* students’ perceptions of the atmosphere, and *SSSP* students’ social self-perceptions

^a^Indicates negatively stated items

### Removal of problematic items

The initial analysis indicated some challenging items, which called for an exploration of how the scales would behave if these challenging items were excluded. In the SPL scale, items 25 and 48 were removed, generating a scalability of *H* = 0.513, including all items in one dimension (lower bound; c = 0.3). When the lower bound was raised to c = 0.4, generating *H* = 0.556, item 47 was appraised as unscalable. For the SPT scale, four items were removed (8, 9, 39, and 50), generating an *H* value of 0.347. The AISP (c = 0.3, which was used for all remaining scales) yielded two dimensions: *H* = 0.400 (items 6, 18, 29, 32, and 37), and *H* = 0.373 (items 2 and 40). Two items (5 and 10) were removed from the SASP subscale, generating *H* = 0.366. The AISP included items 21, 26, 27, 41, and 45 at *H* = 0.412, with item 31 being unscalable at H_i =_ 0.275. Regarding the SPA subscale, items 17 and 35 were removed, generating a scalability coefficient of *H* = 0.343. Two dimensions were formed: *H* = 0.404 (items: 11, 12, 23, 30, 33, 34, 43, and 49), and *H* = 0.366 (items 36 and 42). Two items (4 and 46) were removed from the SSSP subscale, engendering *H* = 0.311. The AISP yielded two dimensions: *H* = 0.417 (items 15, 19, and 28), and *H* = 0.385 (items 3 and 14). The reliability estimates, along with the discarded problematic items, are displayed in Table 2.

## Discussion

The aim of this paper was to introduce the basic principles of MSA, to provide a pragmatic approach for exploring questionnaire data; and to empirically apply MSA to authentic data.

We described the underpinning of MSA and its origin as a non-stochastic, deterministic Guttman scaling method, and its advancement as an analytical method for dichotomous and polytomous items. The fundamental precepts of MSA were addressed, including how MHM and DMM can be used to test whether the data fit the models, as well as the capability of a set of items in contributing toward a common aggregated measure for the ranking of individuals. In concurrence with Watson and colleagues [15], we endeavored in this paper to present the MSA method in a relatively non-mathematical and non-technical way.

### Usefulness of MSA

Many scholars have posited that MSA can offer a detailed and exhaustive analysis of the scalability and dimensionality structure of items, and our findings correspond with those investigators [13, 15, 47]. We argue that anyone who uses or constructs questionnaires, surveys, or tests for measuring attitudes, abilities, personal traits, or opinions in medical education and medical education research will find MSA useful when developing or improving such measurements. Any scale analysis is circuitous, and MSA is no exception. However, we agree with Sijtsma and van der Ark [47] that to portray a comprehensive picture, it is important in the iterative process of MSA to endeavor to assess the assumptions of measurement models as well as to provide quality indices such as scalability and reliability. Our advocated pragmatic and parsimonious approach of using MSA to explore the DREEM instrument revealed no major concerns in the analysis of the item response rate, and neither were the subscales demarcated by considerable floor or ceiling effects. With regard to the subscales, SPL showed moderate scalability, while the scalability for SPA was weak to marginally moderate. However, SPT, SASP, and SSSP exhibited very weak scalability. No major gender differences in scalability were detected. The reversed items allocated to the subscales presented scalability problems. The a priori subscales could not be supported by an explorative AISP, thus resulting in the partitioning of two or three Mokken scales, with the exception of SASP which was not separated. SASP and SSSP displayed *rho* values under 0.70. The results from the unprejudiced exploratory AISP analysis indicate that the five subscales are indeed very close, that “bad” items obscure dimensionality, and that these items can be allocated to more than one of the five subscales. The removal of problematic items from the subscales increased the *H* scalability estimate and generated a *rho* that surpassed the threshold for all subscales except SSSP. Conclusively, our findings seem to be congruent with those of other scholarly studies that have investigated the psychometric properties of DREEM by employing the CTT and PIRT methods, thus suggesting that the instrument is not adequately supported by empirical data [28, 42, 43].

However, it must be highlighted that this paper focuses on the usage and the usefulness of MSA as a non-parametric IRT model, and the DREEM tool is simply used as an example. Thus, using the work of Goffman’s dramaturgical perspectives [48], our empirical results should be viewed from a backstage perspective; the analytical technique proposed by Robert Mokken [14] is the phenomenon of interest and ought to be viewed frontstage. Readers will therefore make their own judgments about the usefulness of NIRT models for their own instruments and in their own context.

#### Strengths and limitations of MSA

MSA offers a thorough exploration of the scalability and dimensionality structure of questionnaire items. It has been posited that NIRT models such as MSA are a very good first step in immediately revealing the most basic characteristics of a questionnaire [6]. By gradually increasing the lower bound c for scalability and thus engaging stronger requirements on the structure of data, MSA can offer alternative ways of forming scales [21]. Reviewing the pattern of cluster outcomes with increasing lower bounds accommodates rich information on the most apt conclusion of scalability and dimensionality.

MSA has some important advantages over CTT: 1) measurement models derived from CTT have an underlying nonrealistic assumption that all items in a questionnaire are equally popular. When this assumption is violated, an artifact can arise whereby items appear not to be abundantly homogeneous to measure a single latent variable. Thus, the MSA model parameters for items also unambiguously recognize that the items vary in popularity and that the analysis lies in the thorough emphasis on model fit. 2) The IRF slopes need to be non-negative [49]. Thus, all *H*_*ij*_ coefficients (consequently, all pairwise relationships) should be positively associated, and items must be appropriately homogeneous with other items. As van Schuur [9] points out, these constraints can harvest instruments that coincide and conform to more persuasive standards of reliability and homogeneity than instruments introspected with conventional CTT reliability analysis. 3) MSA’s “bottom up” clustering technique, which identifies a maximal subset of homogeneous items, is highly practical, especially in explorative phases of a project and during instrument development, and can help identify new presumptive latent variables [9]. 4) MSA is an IRT model that can efficaciously be used for small questionnaire studies and instruments with a small numbers of items [6, 50]. Molenaar [50] has observed that when the number of items is comparatively small, the findings derived from MSA and the more stringent Rasch modeling often generate basically the same results. 5) MSA and its non-parametric IRT models have laid the groundwork for advances of further NIRT models that are different from Guttman’s original cumulative model regarding the specification of their IRF [9, 51].

We also want to further accentuate that MSA also has some leverage over parametric IRT models such as the Rasch model. First, NIRT models employ less restrictive assumptions while still maintaining important measurement properties about the data than most other, often parametric, IRT models [51]. Second, MSA offers valuable tools for exploratory dimensionality analysis that are not easily evaluated in parametric IRT models.

There are also some general drawbacks with MSA. It is much less commonly used than other IRT methods. One reason is that because the IRF is not demarcated parametrically, the person parameters that come out of the IRT cannot be estimated in MSA [8]. It has also been reported that MSA is suitable for investigating scalability but that it is of limited value as a dimensionality assessment method [52]. Roskam et al. [53] have also questioned whether the scaling procedures used in MSA yields ambiguous results. It has also been noted that one disadvantage of using the MSA exploratory item selection procedures to partition items into scales is that the procedure requires scales to be non-overlapping—meaning that items only appear in one scale [54].

### Empirical experiences

Our empirical study also presents some limitations that need to be considered for interpretation. Two major limitations lie in the relatively small number of students and the fact that the study was undertaken in a single context. Straat et al. [55] have highlighted that MSA can detect unidimensional scales with rather small sample sizes and recommend > 250 respondents, if item quality is high, and considerably larger samples if item quality is low. Further, the non-probability sampling method applied may have led to sampling bias, which may have compromised the results. This potential bias may also have been a result of that data was collected both in class and online at a later point in time. Considering the narrowly focused educational measure and the contextual influence of the findings of the real-life data in this study, generalizing beyond physiotherapy students in a traditional Swedish medical university is restricted by the moderate sample size and the singularity of the disciplinary context. However, our intention in the paper was to present the basics of MSA, a powerful method of non-parametric item response theory, and to provide a viable approach and a feasible tool for scholars in medical education research to explore questionnaire data.

Consequently, the empirical study took a pragmatic approach and employed MSA by means of scalability and dimensionality as a first parsimonious step. However, it did not fully investigate violations of the underlying assumptions of the MHM and DMM models. Thus, our analysis of monotonicity and IIO was not entirely comprehensive. As described by Meijer and Egberink [56], it is worth considering that based on our IIO analysis by means of *H*^*T*^ and the visual inspection of plotted item pairs, some “outlying” items—for example, items 9 and 36—may be giving a misleading impression of the strength of IIO. These items have been retained in the present analysis as their removal might have been detrimental to the representation of the underlying constructs that they sought to measure. It can be argued that some features of the underlying assumptions of the NIRT model might be more easily investigated in a parametric model such as the Rasch model. However, in the case of many questionnaires and surveys (e.g., the DREEM), it is often not the intention of constructors to fit items to a particular model, in order to capture an underlying latent trait. Lastly, in our pragmatic approach, we refrained from presenting the uncertainty of the estimated scalability coefficients. However, the standard errors were in the range of 0.030–0.060 for *H*_*i*_ and 0.025–0.030 for *H*. The upper range for *H*_*i*_ was conspicuously evident regarding the reversed items.

### Future perspectives

It has been postulated that MSA is a suitable preliminary step toward evaluating questionnaire data using items with an ordered response level by means of a non-parametric approach [6]. However, one of the authors of the present study (Ulf Brodin) posits a three-step IRT strategy to analyze small-scale questionnaire data [6]. First, to evaluate by means of a non-parametric approach, the set of items must be capable of cooperating with a common aggregated measure, as performed in this study. Further, the secondary step of our material would be to employ the data to a parametric model (e.g., Rasch modelling). Lastly, a third step would be to use a more extended model if required. Thus, a logical secondary step and future perspective would be to employ the data to a parametric IRT model and/or to combine the strength of the IRT psychometric framework with the more established CTT framework. We recommend that scholars in medical education and applied research consider applying non-parametric IRT models to data so as to further understand their ramifications.

## Conclusion

We have presented MSA as a valuable method for exploring the behavior of items in scales in response to varying levels on a latent trait. Our real-data analysis did not provide any strong support for the scalability and dimensional structure of the Swedish version of the DREEM in a sample of undergraduate physiotherapy students.

## Abbreviations

AISP: Automated item selection procedure
CTT: Classical test theory
DMM: Double monotonicity model
DREEM: Dundee Ready Educational Environment Measure
IIO: Invariant item ordering
IRF: Item response function
IRR: Item response rate
IRT: Item response theory
MHM: Monotone homogeneity model
MSA: Mokken scale analysis
MSP: Mokken scaling procedure
NIRT: Non-parametric item response theory
PIRT: Parametric item response theory
SASP: Students’ academic self-perceptions
SPA: Students’ perceptions of atmosphere
SPL: Students’ perceptions of learning
SPSS: Statistical Package for the Social Sciences
SPT: Students’ perceptions of teaching
SSSP: Students’ social self-perceptions
